# Extremes of maternal age and child mortality: analysis between 2000 and 2009☆

**DOI:** 10.1016/j.rpped.2014.05.002

**Published:** 2014-12

**Authors:** Fanciele Dinis Ribeiro, Rosângela Aparecida Pimenta Ferrari, Flávia Lopes Sant'Anna, José Carlos Dalmas, Edmarlon Girotto

**Affiliations:** Universidade Estadual de Londrina (UEL), Londrina, PR, Brazil

**Keywords:** Infant mortality, Maternal age, Risk factors, Causes of death

## Abstract

**OBJECTIVE:**

To analyze the characteristics of infant mortality at the extremes of maternal age.

**METHOD:**

Retrospective, cross-sectional quantitative study using data from Live Birth Certificates, Death Certificates and from Child Death Investigation records in Londrina, Paraná, in the years of 2000-2009.

**RESULTS:**

During the 10-year study period

**,:**

there were 176 infant deaths among mothers up to 19 years of age, and 113 deaths among mothers aged 35 years or more. The infant mortality rate among young mothers was 14.4 deaths per thousand births, compared to 12.9 deaths in the other age group. For adolescent mothers, the following conditions prevailed: lack of a stable partner (*p*<0.001), lack of a paid job (*p*<0.001), late start of prenatal care in the second trimester of pregnancy (*p*<0.001), fewer prenatal visits (*p*<0.001) and urinary tract infections (*p*<0.001). On the other hand, women aged 35 or more had a higher occurrence of hypertension during pregnancy (*p*<0.001), and of surgical delivery (*p*<0.001). Regarding the underlying cause of infant death, congenital anomalies prevailed in the group of older mothers (*p*=0.002), and external causes were predominant in the group of young mothers (*p*=0.019).

**CONCLUSION:**

Both age groups deserve the attention of social services for maternal and child health, especially adolescent mothers, who presented a higher combination of factors deemed hazardous to the child's health.

## Introduction

Maternal age is considered one of the determinants of gestational risk. Thus, mothers aged younger than 15 years or whose menarche happened less than two years before pregnancy, and mothers aged 35 years or older have higher risk of neonatal death. Other gestational risk factors also found in the literature are unfavorable sociodemographic conditions, previous reproductive history and preexisting clinical conditions.^1^

Worldwide, approximately 25% of women have their first child before the age of 20, a percentage that is higher in developing countries.^2^ In adolescence, pregnancy occurs due to early sexual initiation, low self-esteem and/or lack of inclusion in social projects. Unplanned pregnancies in this age group generate increased school dropout rates and underemployment, psychosocial risk, inadequate termination of pregnancy and non-adherence to prenatal care.^1^^-^ 5

On the other hand, pregnancy in women older than 35 years is an observable trend in developed and developing countries, including Brazil.^4^^,^ 5 Among the reasons, we highlight the changing social role of women in the labor market and within the family, the search for financial stability and the achievement of high educational levels. The social, economic and emotional maturity results in greater knowledge about the importance of adequate monitoring of pregnancy; however, they can become risk factors for late pregnancy.^1^

In Brazil, in 2010, there was a total of one million pregnant adolescents out of the 17 million registered ones. As for mothers aged 30 or older, there was a 21.0% increase in births between 2000 and 2010. In this period, the percentage of women aged 35 to 39 years that had children increased from 6.7% to 8.0% in the country, while the percentage of mothers aged 40 years increased from 1.9% to 2.3%.^5^^-^ 6 There are different associations that lead to a higher probability of death among children born to very young, as well as older women, before their first year of life.^2^ In terms of early motherhood, the discussions comprehend the physiological immaturity, especially uterine, that leads to intrauterine growth restriction, low birth weight and prematurity, as well as the unfavorable ​​socioeconomic situation and the psychological fragility. On the other hand, mothers older than 35 years have a higher risk of morbidities during pregnancy, such as diabetes mellitus and hypertension, with repercussions to the fetus and the newborn, as well as an increased incidence of diseases of genetic origin, miscarriages and multiple pregnancies resulting from assisted fertilization.^7^^-^ 9

Thus, considering that motherhood in the extremes of maternal age is a complex event that might involve risks to the healthy development of the child, this study aimed to analyze the characteristics of infant death in the extremes of maternal age. 

## Method

This is a retrospective, cross-sectional study carried out in Londrina, Paraná, from 2000 to 2009. The study population consisted of mothers at the extremes of maternal age, i.e., up to 19 years old and 35 years or older, whose children died before reaching one year of age. Data collection occurred from March to July 2010, based on the Live Birth Notifications (LBNs), Death Certificates (DCs) and Infant Death Investigation records of the Municipal Committee for the Prevention of Child-Maternal Mortality (CMPMMI), filed in the Mortality Information Center (NIM) of the Municipal Health Secretariat. 

The NIM is a sector of the Health Information Management, which aims to investigate the real causes of mortality in Londrina residents, improve the quality of information on mortality, and streamline and democratize access to information for health services and the population. Therefore, it provides subsidies to plan and prioritize health actions aimed at reducing premature and/or preventable deaths. 

During the 10 years considered in this study, 783 infant deaths occurred. To achieve the desired sample, we excluded children born to mothers aged between 20 and 34 years, totaling 289 children who died before completing the first year of life. Out of these, 176 (60.9%) had adolescent mothers with a minimum age of 10, and a maximum age of 19, and 113 (39.1%) had mothers aged 35 or older. 

The variables were grouped into: extremes of age - teenagers (10-19 years) and women with advanced age (35 and older); sociodemographic status - marital status (with partner, without partner), education (<8 years and ≥ 8 years of schooling), income (<2 and ≥ 2 minimum wages per family), occupation (paid and unpaid); prenatal care - received prenatal care (yes and no), start of prenatal care (1^st^, 2^nd^ and 3^rd^ trimester), number of prenatal consultations (<7 and ≥ 7), maternal-fetal problems/diseases during pregnancy - preterm labor (PTL) (yes and no), urinary tract infection (UTI) (yes and no), premature rupture of membranes (PROM) (yes and no), arterial hypertension/ pre-eclampsia (SAH) (yes or no); delivery and birth characteristics - gestational age (<37 and ≥ 37 weeks), type of delivery (vaginal and surgical) and hospital discharge (yes and no); characteristics of the newborn - birth weight (<2,500 and ≥ 2,500 grams), Apgar scores at 1 and 5 minutes (<7 and ≥ 7); characteristics of infant death - year of death (in biennia), basic cause - perinatal conditions (yes and no), congenital abnormalities (yes and no), external causes (yes and no), respiratory diseases (yes or no) and period of death (0-27 days=neonatal and 28-364 days=post-neonatal). 

Data were collected on a previously tested form, and typed in double entry in the Epi Info(r) program. Statistical analysis was carried out with the SPSS(r) program, considering the chi-square test or Fisher's exact test, with a significance level of 5% (*p*<0.05). To calculate the infant mortality rate by extremes of maternal age, the numerator consisted of the total infant deaths, and the denominator, the total number of live births, both by maternal age range. 

The study was submitted to the analysis of the Department of Health Care of the Municipal Health Authority of Londrina and the Research Ethics Committee of *Universidade Estadual de Londrina* CEP / UEL, being approved under the number CAAE 01079112.3.0000.5231. 

## Results

The mean age of the adolescent mothers was 17 years, and of those aged 35 and over, 36 years. During the years 2000 to 2009, 72,012 living children were born in Londrina, with the largest concentration of births in 2000 (8,368), and the lowest in 2007 (6,750). The infant mortality rate in the municipality during the ten years studied combined was 10.9 deaths per 1,000 live births. When separated by extremes of maternal age, the rate was higher among adolescent mothers, at 14.4 deaths per 1,000 live births, compared to mothers aged >35 years (12.9 deaths per 1,000 live births). 

**Figure 1:**
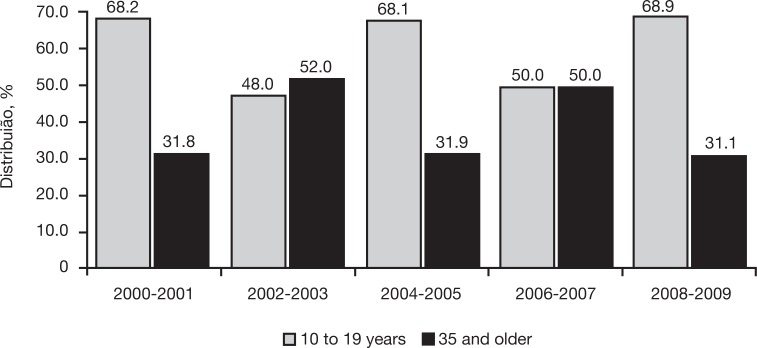
Distribution of the extremes of maternal age per biennium, 2000 to 2009, Londrina (PR)

Fig. 1 shows that, in three of the five analyzed biennia, there was a higher frequency of infant death in the group of mothers aged 10-19, with a percentage ranging from 68.1% (2004-2005) to 68.9% (2008-2009). This proportion was higher among women aged 35 or more (52.0%) only in the 2002-2003 biennium.

Table 1 shows maternal sociodemographic characteristics. An income up to the minimum wage was more frequent among adolescents (37.0%) when compared to mothers aged 35 or more (*p*=0.052). Approximately 31.0% of the adolescents had no partner, and 80.6% did not have a paid job. In contrast, more than half of women aged 35 years or more had a partner (86.7%) and a paying job (52.2%) (*p*<0.001). 

**Table 1:**
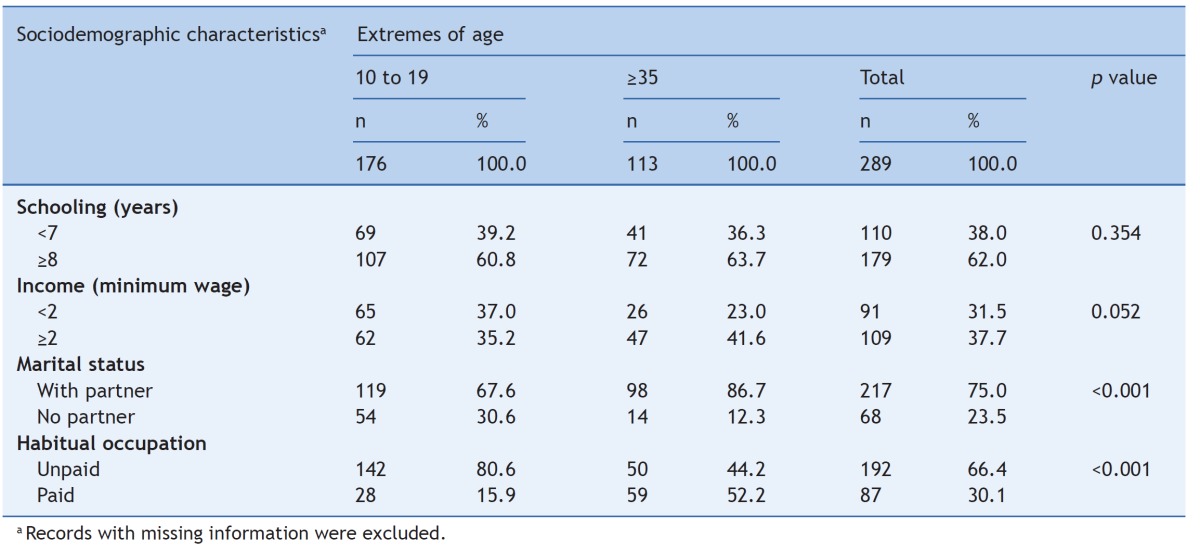
Sociodemographic characteristics and extremes of maternal age (years), 2000 to 2009, Londrina (PR).

As for the women's obstetric history, prenatal follow-up was performed by more than 90.0% in both age groups. The start of follow-up predominated in the first trimester (65.0%), but the percentage was higher (76.1%) among those aged 35 and older, when compared to adolescent mothers (57.9%) (*p*<0.001). None of the women started prenatal care in the third trimester of pregnancy (Table 2). Regarding the number of pre-natal consultations, Table 2 shows that only 27.2% of the adolescents had seven or more, while in the other age group this rate was higher (53.9%, *p*=0.001). The presence of diseases during pregnancy was outstanding in both age groups, with the most common being preterm labor, premature rupture of membranes, urinary tract infections and systemic arterial hypertension. It was observed that both preterm labor and premature rupture of membranes affected both age groups similarly, with no statistical significance. In contrast, urinary infection was more frequent in women up to 19 years, and systemic arterial hypertension was more frequent among those aged 35 or older, with *p*<0.001. 

**Table 2:**
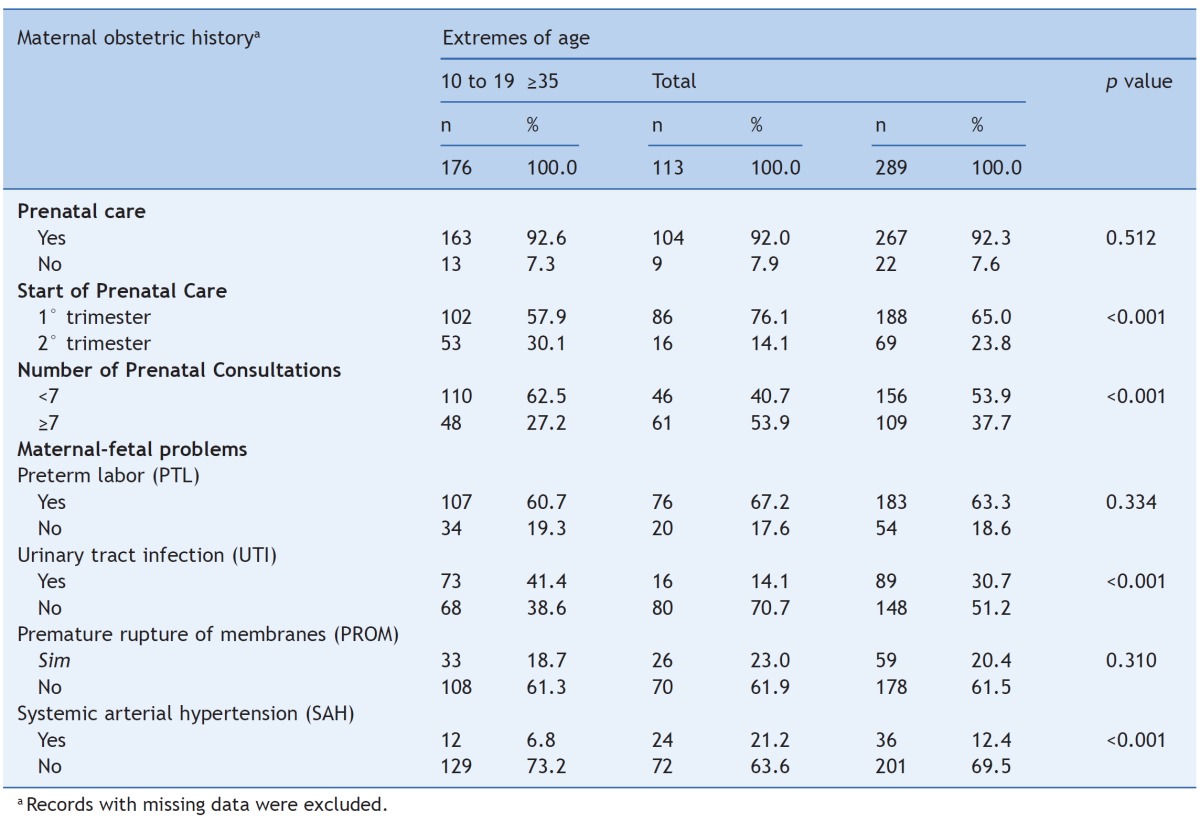
Maternal and obstetric history and extremes of age (in years), 2000 to 2009, Londrina (PR).

Regarding the type of delivery, Table 3 shows that surgical delivery predominated among older women (58.4%), while vaginal delivery predominated among adolescents (61.9%) (*p*<0.001). Prematurity, low birth weight, low Apgar score at 1 minute and Apgar score ≥ 7 at 5 minutes were similar characteristics in newborns of pregnant women in both age groups, with no statistical significance. The percentage of hospital discharge after birth did not reach 30.0%, as 70.0% of children who died in the first year of life died in the neonatal period in both groups analyzed. 

**Table 3:**
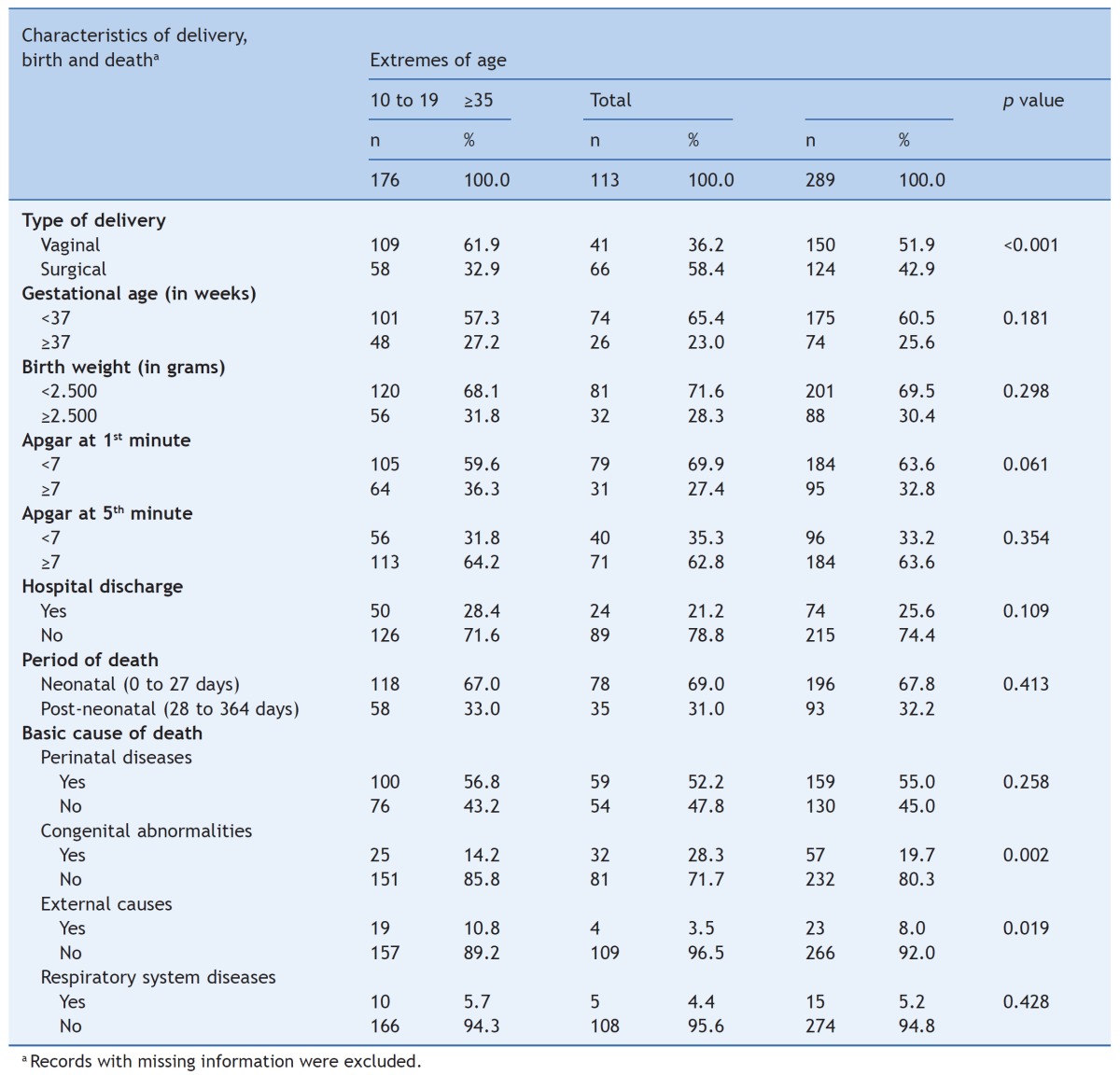
C Characteristics of type of delivery, birth and death between the extremes of maternal age (years), 2000 to 2009, Londrina, PR.

The most common underlying cause of death was perinatal condition in both maternal age groups. Congenital anomalies were twice as common among women aged 35 or older. In contrast, external causes affected three times more often the children of adolescent mothers (Table 3), with the main one being the aspiration of gastric contents at home. 

## Discussion

The effects of maternal age on child health have been the subject of extensive discussions in the literature, ranging from changes in the role of women in contemporary society to risks for fetal and neonatal health.^9^ In Brazil, there has been a gradual decline in fertility. In 2000, the mean number of children per woman was 2.38, which decreased to 1.9 in 2010, a reduction of 20.1%. The Northern region has the highest fertility rate, 2.51 children per woman. The Northeast, Midwest and South regions show fertility rates of 2.04, 1.93 and 1.92, respectively. As for the Southeast region, it had the lowest national mean (1.75).^6^^,^ 10 Brazilian women are waiting longer to have children, and despite the increase in the number of teenage pregnancies, the median fertility age increased from 26.3 years in 2000 to 26.8 years in 2010. The fertility among women aged 15 to 19 decreased from 18.8% in 2000 to 17.7% in 2010. Among those aged 20-24, it went from 29.3% in 2000 to 27.0% in 2010. In addition to these reductions, there was also a decrease in infant deaths, from 29.7 to 15.6 per thousand live births, a decrease of 47.6 % between 2000 and 2010.^5^^,^ 6

In this study, it was observed that in almost all biennia, infant deaths were more frequent among women aged 10 to 19. In addition, the infant mortality rate among adolescents was higher when compared to mothers of advanced reproductive age. Data from the Ministry of Health showed that, out of the 42,684 children younger than one year that died in 2009, 7,917 were born to women aged between 15 and 19, and 627 were born to adolescents aged <14. Together, these deaths accounted for 20.0% of all infant deaths.6 Another study found that 27.1% of neonatal deaths occurred among adolescent mothers.^10^

Maternal educational level in this study was not statistically significant between the extremes of age, contrary to what is shown in the literature.^11^ Achieving high educational levels may represent a stimulus for the postponement of the birth of the first child, because the higher the educational level, the more likely it will be that the first intercourse will not occur prematurely; marriage will be postponed; the use of contraceptive methods will be higher, and the formation of smaller families will be favored.^9^^,^ 12 ^,^ 13

On the other hand, lower income, being unmarried and having no paid work were more frequent factors in the group of women aged 10 to 19, when compared to those aged 35 or older. Studies carried out with adolescents showed that the number of formal marital unions is small, and new families are formed within the same physical space, i.e., the new family lives with the family of origin of one of the spouses.^9^^,^ 12 ^-^ 14 On the other hand, IBGE (Brazilian Institute of Geography and Statistics) data indicate that formal unions were more common among pregnant women aged 30 or more.^6^ These differences highlight the peculiarities of each phase, from the psychoaffective immaturity and socioeconomic fragilities of adolescent mothers, to postponement of motherhood caused by the greater inclusion of women in the labor market, as well as investing in the career to improve financial condition among mothers of advanced age.^9^^,^ 15 ^-^ 18

Regarding the information about prenatal care, almost all women in the two extremes of age received prenatal care in this study, but a lower frequency of start of prenatal care in the first trimester and lower number of consultations occurred in the group of adolescent mothers, corroborating studies that reported that this age group seeks prenatal care at a later time, and thus, does not have complete and adequate care.^3^^,^ 12 ^,^ 15 ^,^ 19 The higher number of consultations during pregnancy at the extremes of maternal age has been considered as a condition to reduce the incidence of maternal and fetal complications, especially in adolescence, as these pregnant women seek health care services less frequently.^1^^,^ 3 ^,^ 12 ^,^ 15 ^,^ 20

Systematic prenatal follow-up does not occur only with the minimum number of consultations recommended by the Ministry of Health. Interdisciplinary and multidisciplinary support to the pregnant women and their families is also necessary, so that there is early detection of gestational diseases and timely treatment to prevent risks to the mother-child binomial, mainly genitourinary tract infections and cardiovascular and metabolic disorders.^1^ Most of these conditions result in preterm delivery, low birth weight, PROM and sepsis.^8^^,^ 12 ^,^ 15 ^,^ 19 ^-^ 22 In this study, urinary tract infections were more frequent in mothers aged up to 19, and hypertension was more frequent among those aged 35 or older. It is noteworthy that urinary infections are seen as an inducer of premature labor, resulting in severe complications for the newborn, not only due to the infant's immature body, but also due to infectious disorders caused by these infections. As for systemic arterial hypertension during pregnancy, it is more frequent among older women, and it is considered one of the risk factors for neonatal death, as well as metabolic diseases and congenital anomalies.^7^^,^ 15 ^,^ 18 ^,^ 21 ^,^ 23

Regarding the surgical delivery, the literature indicates that this is more common among women older than 35 years.^8^^,^ 16 ^,^ 19 These data were also observed in the present study. The indication for surgical delivery is convenient for women at obstetric risk; however, it has frequently been indicated to low-risk pregnant women with higher income and education, with seven or more prenatal consultations, and who are treated in the private healthcare network, exposing the infant to iatrogenic disorders resulting from this process.^21^^-^ 23

As for the birth conditions of children younger than one year in this study, prematurity occurred at both extremes of maternal age and showed no statistically significant association. In contrast, some studies found that adolescents are 75% more likely to have a premature delivery than adult women.^19^^,^ 24 However, other studies indicated that late motherhood shows an increased occurrence of low birth weight, preterm delivery, neonatal asphyxia, chromosomal abnormalities and macrosomias.^12^^,^ 13 A study carried out in Liverpool with 9,506 birth records showed a higher prevalence of premature birth and low birth weight in the two extremes of age, adolescent mothers and those aged over 40.^18^ Low birth weight is an isolated indicator of child morbimortality,^19^^,^ 22 ^,^ 25 and its incidence when compared to that among adult women is more than twice than that among adolescents, and two times higher among women aged 35 or older.^8^

In the present study, few children were discharged after birth, and the highest proportion of deaths occurred within the first 27 days of life. The neonatal mortality suggests causes arising especially from poor health care offered to mothers during the prenatal period and childbirth, as well as to the newborns in the delivery room and neonatal units. In such cases, it is necessary to implement strategies in the perinatal field with actions directed to the development and improvement of maternal and child health services.^26^^,^ 27

Another characteristic observed was the predominance of perinatal diseases as the cause of death, followed by congenital abnormalities and external causes. In a study carried out in the municipality of Cianorte, Paraná between 1999 and 2006, it was observed that 80.7% of infant deaths occurred during the neonatal period, and 68.3% were due to perinatal causes.^12^ The high proportions of death by perinatal conditions are warning signs, as they may indicate early termination of pregnancy or intrauterine retarded growth, the result of inadequate prenatal care.^21^^,^ 23 ^,^ 27 As for congenital abnormalities, studies indicate a higher incidence among women of advanced age,^7^^,^ 10 ^,^ 12 ^,^ 18 ^,^ 21 ^,^ 23 corroborating the findings of this study. In contrast, external causes were more evident in the children of adolescent mothers, a fact of concern as they are preventable events that may be associated with the lack of preparedness on the part of young mothers to care for their children.

Most infant deaths in Brazil can be considered preventable, if adequate and timely access to health services is guaranteed. Therefore, investments in qualified human resources in the assistance of both mother and child in all instances of care, as well as the presence of a hierarchized, effective physical structure of care can minimize the occurrence of these deaths.^1^^,^ 21 ^,^ 23 ^-^ 25

Therefore, we emphasize the need for greater investment in the prevention and promotion of maternal health, especially in the extremes of maternal age and particularly for adolescents, seeking to reduce gestational diseases and preventable infant deaths. Longitudinal studies that specifically work with extremes of maternal age should be encouraged to better elucidate these findings and contribute to the existing literature.
