# Supplementary material for: Extremes of maternal age and child mortality: analysis between 2000 and 2009
Source: Rev Paul Pediatr. 2014 Dec;32(4):381–8. doi: 10.1016/j.rpped.2014.05.002 (PMC4311793; doi:10.1016/j.rpped.2014.05.002)
Supplement: Translation

# Translation: Extremos de idade materna e mortalidade infantil: análise entre 2000 e 2009☆

**Authors:** Fanciele Dinis Ribeiro, Rosângela Aparecida Pimenta Ferrari, Flávia Lopes Sant'Anna, José Carlos Dalmas, Edmarlon Girotto

**Affiliations:** Universidade Estadual de Londrina (UEL), Londrina, PR, Brasil

**Keywords:** Mortalidade infantil, Idade materna, Fatores de risco, Causas de morte

## Abstract

**OBJETIVO:**

Analisar as características do óbito infantil nos extremos de idade materna.

**MÉTODO:**

Estudo quantitativo, do tipo retrospectivo e transversal, utilizando dados da Declaração de Nascido Vivo, Declaração de Óbito e Ficha de Investigação do Óbito Infantil em Londrina, Paraná, nos anos de 2000 a 2009.

**RESULTADOS:**

Nos dez anos de estudo, houve 176 óbitos infantis de mães com até 19 anos, e 113 de mães com 35 anos ou mais. A taxa de mortalidade infantil entre as mães jovens foi de 14,4 mortes a cada mil nascimentos, comparado a 12,9 mortes no outro grupo etário. Para as mães adolescentes, prevaleceu situação conjugal sem companheiro (*p*<0,001), não exercer atividade remunerada (*p*<0,001), início tardio do pré-natal no segundo trimestre de gestação (*p*<0,001), menor número de consultas pré-natais (*p*<0,001) e infeções urinárias (*p*<0,001). Por outro lado, as mulheres com 35 anos ou mais tiveram maior frequência de hipertensão arterial durante a gestação (*p*<0,001) e de parto cirúrgico (*p*<0,001). Com relação à causa básica do óbito infantil, as anomalias congênitas predominaram no grupo de mães com idade avançada (*p*=0,002), e as causas externas, no grupo de mães jovens (*p*=0,019).

**CONCLUSÃO:**

Ambos os grupos etários merecem atenção dos serviços assistenciais de saúde materna e infantil, especialmente as mães adolescentes que agregaram maior conjunto de fatores considerados de risco à saúde da criança.

## Introdução

A idade materna é considerada um dos determinantes de risco gestacional. Assim, mães com idade inferior a 15 anos ou com menarca há menos de dois anos e mães com idade igual ou superior a 35 anos levam a maior risco de óbito neonatal. Outros fatores de risco gestacional também encontrados na literatura são: condições sociodemográficas desfavoráveis, história reprodutiva anterior e condições clínicas preexistentes.^1^

No mundo, aproximadamente 25% das mulheres tem seu primeiro filho antes de completar 20 anos de idade, percentual que é maior nos países em desenvolvimento.^2^ Na adolescência, a maternidade ocorre pela iniciação sexual precoce, baixa auto-estima e/ou falta de inclusão em projeto social. A gravidez não planejada nesse grupo etário gera aumento dos índices de abandono escolar e subemprego, risco psicossocial, interrupção indevida da gestação e não-adesão ao acompanhamento pré-natal.^1^^-^ 5

Já a maternidade a partir dos 35 anos é uma tendência observável em países desenvolvidos e em desenvolvimento, incluindo o Brasil.^4^^,^ 5 Entre os motivos destacam-se a mudança do papel social da mulher no mercado de trabalho e dentro do núcleo familiar, busca de estabilidade financeira e alcance de níveis educacionais elevados. O amadurecimento social, econômico e afetivo proporciona maior esclarecimento sobre a importância do acompanhamento adequado da gestação, entretanto podem tornar-se fatores de risco para a gravidez tardia.^1^

No Brasil, em 2010, a gravidez ocorreu em um milhão de adolescentes das 17 milhões registradas. Quanto às mães com 30 anos ou mais, entre 2000 e 2010, houve aumento de 21,0% nos nascimentos. Neste período, a porcentagem de mulheres que tiveram filhos com idade entre 35 e 39 anos passou de 6,7% para 8,0% no país, enquanto o percentual de mães com 40 anos subiu de 1,9% para 2,3%.^5^^-^ 6 Tanto para mães muito jovens, quanto para mães em idade avançada, há diferentes associações que conduzem à maior probabilidade de morte dos filhos antes de completarem o primeiro ano de vida.^2^ No que se refere à maternidade precoce, as discussões abarcam a imaturidade fisiológica, principalmente uterina, que levam à restrição do crescimento intrauterino, baixo peso ao nascer e prematuridade, além de condições socioeconômicas desfavoráveis e fragilidade psicológica. Por outro lado, mães com mais de 35 anos possuem maior risco de morbidades durante a gestação, como diabetes melito e hipertensão arterial, com repercussões ao feto e recém-nascido, além de maior incidência de afecções de origem genética, abortos espontâneos e gestações múltiplas decorrentes de fertilização assistida.^7^^-^ 9

Nesse sentido, considerando que a maternidade nos extremos de idade é complexa e pode implicar em riscos ao desenvolvimento saudável da criança, o presente estudo teve como objetivo analisar as características do óbito infantil nos extremos de idade materna.

## Método

Trata-se de um estudo retrospectivo e transversal, realizado em Londrina, Paraná, no período de 2000 a 2009. A população foi composta por mães em extremos de idade, ou seja, até 19 anos e maior ou igual a 35 anos, cujos filhos faleceram antes de completar um ano de vida. A coleta de dados ocorreu nos meses de março a julho de 2010 a partir da Declaração de Nascido Vivo (DNV), da Declaração de Óbito (DO) e das Fichas de Investigação de Óbito Infantil do Comitê Municipal de Prevenção de Mortalidade Materna Infantil (CMPMMI), arquivada no Núcleo de Informação de Mortalidade (NIM) da Secretaria Municipal de Saúde. 

O NIM é um setor da Gerência de Informações em Saúde que tem como objetivo pesquisar as reais causas de mortalidade dos residentes em Londrina, aprimorar a qualidade da informação em mortalidade e democratizar e agilizar o acesso à informação aos serviços de saúde e população. Com isso, fornece subsídios para planejar e priorizar as ações de saúde, visando reduzir as mortes prematuras e/ou evitáveis. 

Durante os 10 anos considerados neste estudo, ocorreram 783 óbitos infantis. Para alcance da casuística desejada, foram excluídos filhos de mães com idades entre 20 e 34 anos, totalizando 289 crianças que morreram antes de completar o primeiro ano de vida. Dessas, 176 (60,9%) eram de mães adolescentes com idade mínima de 10 anos e máxima de 19 anos, e 113 (39,1%) eram de mães com 35 anos ou mais. 

As variáveis foram agrupadas em: *extremos de idade - *adolescentes (10 a 19 anos) e mulheres com idade avançada (35 anos e mais); *condições sociodemográficas* - situação conjugal (com companheiro e sem companheiro), escolaridade (<8 e ≥8 anos), renda (<2 salários mínimos e ≥2 salários mínimos por família), ocupação (remunerada e não remunerada); acompanhamento pré-natal - realização do pré-natal (sim e não), início do pré-natal (1°, 2° e 3º trimestre), número de consultas pré-natais (<7 e ≥7), problemas/doenças materno-fetais na gestação - trabalho de parto prematuro (TPP) (sim e não), infecção das vias urinárias (ITU) (sim e não), rotura prematura de membranas (ROPREMA) (sim e não), hipertensão arterial/pré-eclampsia (HAS) (sim e não); características do nascimento e parto - idade gestacional (<37 e ≥37 semanas), tipo de parto (vaginal e cirúrgico) e alta hospitalar (sim e não); características do recém-nascido - peso ao nascer (<2500 e ≥2.500 gramas), Apgar no 1º e 5º minutos (<7 e ≥7); *características do óbito infantil* - ano do óbito (em biênios), causa básica - afecções perinatais (sim e não), anomalias congênitas (sim e não), causas externas (sim e não), doenças do aparelho respiratório (sim e não), e período do óbito (0 a 27 dias = neonatal e 28 a 364 dias = pós-neonatal).

Os dados foram coletados em formulário previamente testado, sendo digitados em dupla entrada no programa Epi Info^(r)^. Para análise estatística foi utilizado o programa SPSS^(r)^, considerando o teste qui-quadrado ou teste exato de Fisher, com nível de significância de 5% (*p*<0,05). Para o cáculo do coeficiente de mortalidade infantil por extremos de idade materna, utilizou-se no numerador o total de óbitos infantis, e no denominador o total de nascidos vivos, ambos por faixa etária materna. 

A pesquisa foi submetida à apreciação da Diretoria de Atenção à Saúde da Autarquia Municipal de Saúde de Londrina e do Comitê de Ética em Pesquisa da Universidade Estadual de Londrina CEP/UEL, tendo recebido parecer favorável sob o número CAAE 01079112.3.0000.5231. 

## Resultados

A média de idade das mães adolescentes foi de 17 anos e daquelas com 35 anos e mais, de 36 anos. Durante os anos 2000 a 2009 nasceram em Londrina 72.012 crianças vivas, com concentração do maior número de nascimentos no ano 2000 (8.368), e do menor em 2007 (6.750). A taxa de mortalidade infantil no município nesses dez anos estudados, em conjunto, foi de 10,9 mortes por mil nascidos vivos. Quando separados por extremos de idade materna, a taxa foi maior entre mães adolescentes com 14,4 mortes por mil nascidos vivos, em relação àquelas com idade igual ou maior que 35 anos (12,9 mortes por mil nascidos vivos).

Observa-se na figura 1 que, em três dos cinco biênios analisados, houve maior frequência de óbito infantil no grupo de mães de 10 a 19 anos de idade, com percentual variando de 68,1% (2004-2005) a 68,9% (2008-2009). Somente no biênio 2002 a 2003 esta proporção foi superior nas mulheres com 35 anos ou mais (52,0%).

**Figura 1:** Distribuição dos extremos de idade materna por biênio, 2000 a 2009, Londrina, PR

Na tabela 1 encontram-se as características sociodemográficas maternas. A renda de até um salário mínimo foi mais frequente entre as adolescentes (37,0%) comparadas às mães de 35 anos ou mais (*p*=0,052). Aproximadamente 31,0% das adolescentes não tinham companheiro e 80,6% não exercíciam atividade remunerada. Ao contrário, mais da metade das mulheres com 35 anos ou mais tinham companheiro (86,7%) e atividade remunerada (52,2%) (*p*<0,001). 

**Tabela 1:** Características sociodemográficas e extremos de idade materna (em anos), 2000 a 2009, Londrina, PR

Quanto à história obstétrica das mulheres, o seguimento pré-natal foi realizado por mais de 90,0% em ambas as faixas de idade. O início do acompanhamento predominou no 1º trimestre (65,0%), mas aquelas com 35 anos ou mais apresentaram maior percentual (76,1%), comparado às mães adolescentes (57,9%) (*p*<0,001). Nenhuma das mulheres iniciou o pré-natal no 3º trimestre de gestação (tabela 2). No que diz respeito ao número de consultas no pré-natais, observa-se ainda na tabela 2 que somente 27,2% das adolescentes fizeram sete ou mais consultas, enquanto no outro grupo etário essa frequência foi maior (53,9%; *p*=0,001). A presença de afecções durante a gestação foi marcante em ambas as idades, sendo as mais comuns o trabalho de parto prematuro, a rotura prematura de membranas, a infecção do trato urinário e a hipertensão arterial sistêmica. Verificou-se que tanto o trabalho de parto prematuro como a rotura prematura de membranas acometeram ambas as idades de forma semelhante, não havendo significância estatística. Ao contrário, a infecção urinária foi mais frequente nas mulheres com até 19 anos e a hipertensão arterial entre aquelas com 35 anos ou mais, com *p*<0,001.

**Tabela 2:** História obstétrica materna e extremos de idade (em anos), 2000 a 2009, Londrina, PR

Com relação ao tipo de parto, a tabela 3 mostra que o cirúrgico prevaleceu entre as mulheres de mais idade (58,4%), enquanto o vaginal predominou entre as adolescentes (61,9%) (*p*<0,001). A prematuridade, o baixo peso ao nascer, o baixo índice de Apgar no 1º minuto e Apgar ≥7 no 5º minuto foram características similares em recém-nascidos de gestantes de ambas as faixas etárias, sem significância estatística. O percentual de alta hospitalar após o nascimento não chegou a 30,0%, uma vez que 70,0% das crianças que morreram no primeiro ano de vida faleceram ainda no período neonatal nos dois grupos analisados.

**Tabela 3:** Características do parto, nascimento e óbito entre os extremos de idade materna (em anos), 2000 a 2009, Londrina, PR

A causa básica do óbito mais frequente foi afecção perinatal em ambas as idades maternas. As anomalias congênitas foram duas vezes mais frequentes entre as mulheres com 35 anos ou mais. Ao contrário, as causas externas acometeram três vezes mais os filhos das adolescentes (tabela 3), sendo a principal delas a aspiração de conteúdo gástrico em domicílio. 

## Discussão

Os efeitos da idade materna sobre a saúde da criança contam com amplas discussões na literatura, que vão desde mudanças no papel da mulher na sociedade contemporânea até riscos sobre a saúde fetal e neonatal.^9^ No Brasil há gradativa redução da fecundidade. Em 2000, a média de filhos por mulher era 2,38 e passou para 1,9 em 2010, queda de 20,1%. A região Norte detém a maior taxa de fecundidade, 2,51 filhos por mulher. As regiões Nordeste, Centro-Oeste e Sul apresentam, respectivamente, taxa de fecundidade de 2,04, 1,93 e 1,92. Já a região Sudeste teve a menor média nacional (1,75).^6^^,^ 10 As mulheres brasileiras estão esperando mais tempo para ter filhos e, apesar do aumento no número de adolescentes grávidas, a idade média de fecundidade passou de 26,3 anos, em 2000, para 26,8 anos, em 2010. A fecundidade entre mulheres com 15 a 19 anos diminuiu de 18,8%, em 2000, para 17,7%, em 2010. Já entre aquelas com 20 a 24 anos de 29,3%, em 2000, para 27,0%, em 2010. Além dessas reduções, também houve dimuinuição das mortes infantis, passando de 29,7 para 15,6 por mil nascidos vivos, uma queda de 47,6%, entre 2000 a 2010.^5^^,^ 6

Neste estudo verificou-se que, em quase todos os biênios, os óbitos infantis foram mais frequentes entre mulheres na faixa etária dos 10 aos 19 anos. Ademais, a taxa de mortalidade infantil entre adolescentes foi maior comparada às mães com idade reprodutiva avançada. Dados do Ministério da Saúde apontaram que, em 2009, das 42.684 crianças menores de um ano que tiveram como desfecho o óbito, 7.917 eram filhas de mulheres entre 15 e 19 anos e 627 de adolescentes com menos de 14 anos. Somados, esses óbitos corresponderam a 20,0% do total de mortes infantis.^6^ Outro estudo identificou que 27,1% dos óbitos neonatais ocorreram entre mães adolescentes.^10^

A escolaridade materna no presente estudo não se mostrou estatisticamente significativa entre os extremos de idade, contrariando ao apontado na literatura.^11^ O alcance de níveis educacionais elevados pode representar um estímulo para o adiamento do nascimento do primeiro filho, pois, quanto maior a escolaridade, maior a tendência de que a primeira relação sexual não aconteça precocemente, que a entrada no casamento seja postergada, que o uso de métodos contraceptivos seja maior e que se valorize a constituição de famílias menores.^9^^,^ 12 ^,^ 13

Por outro lado, a menor renda, estar sem companheiro e não ter atividade remunerada foi mais frequente no grupo das mulheres entre 10 e 19 anos comparado àquelas com 35 anos ou mais. Estudos realizados com adolescentes revelaram que é pequeno o índice de casamentos formais e que novos núcleos familiares formam-se num mesmo espaço físico, ou seja, a nova família passa a morar com a família de origem de um dos cônjuges.^9^^,^ 12 ^-^ 14 Por outro lado, dados do IBGE apontam que a união formal esteve mais presente entre as gestantes com idade acima de 30 anos.^6^ Essas diferenças evidenciam as peculiaridades de cada fase, desde imaturidade psicoafetiva e fragilidades socioeconômicas características de mães adolescentes, até postergação da maternidade pela maior inserção da mulher no mercado de trabalho, além de investimentos na carreira profissional para melhorar a condição financeira entre mães com idade mais avançada.^9^^,^ 15 ^-^ 18

Quanto às informações sobre o acompanhamento pré-natal, quase a totalidade, nos dois extremos de idade, realizaram o seguimento neste estudo, mas a menor frequência de início ainda no primeiro trimestre e de consultas ocorreu no grupo de mães adolescentes, corroborando com estudos que referem que tal grupo etário tarda em procurar assistência pré-natal e acaba não tendo um acompanhamento completo e adequado.^3^^,^ 12 ^,^ 15 ^,^ 19 O maior número de consultas durante a gestação nos extremos de idade tem sido considerado como condição para reduzir a incidência de complicações materno-fetais, em especial na adolescência, visto que essas gestantes buscam menos os serviços assistenciais.^1^^,^ 3 ^,^ 12 ^,^ 15 ^,^ 20

O acompanhamento sistemático no programa pré-natal não ocorre somente com o número mínimo de consultas preconizadas pelo Ministério da Saúde. Há necessidade de apoio interdisciplinar e multiprofissional à gestante e sua família para que haja detecção precoce de doenças gestacionais e tratamento em tempo oportuno para prevenir riscos ao binômio advindos, principalmente, das infecções do trato genitourinário, distúrbios cardiovasculares e metabólicos.^1^ A maioria dessas afecções resulta em parto pré-termo, baixo peso, roprema e sepse.^8^^,^ 12 ^,^ 15 ^,^ 19 ^-^ 22 Neste estudo a infecção de trato urinário foi mais frequente em mães com até 19 anos e a hipertensão arterial entre aquelas com 35 anos ou mais. Ressalta-se que a infecção urinária é vista como indutora do trabalho de parto prematuro, trazendo graves complicações ao neonato não apenas pelo seu organismo imaturo, mas também pelos transtornos infecciosos dela decorrentes. Já a hipertensão durante a gestação tem sido mais frequente entre mulheres em idade avançada, e é apontada como um dos fatores de risco para a morte neonatal, bem como doenças metabólicas e anomalias congênitas.^7^^,^ 15 ^,^ 18 ^,^ 21 ^,^ 23

No que se refere ao parto cirúrgico, a literatura aponta que este é mais frequente em mulheres com mais de 35 anos de idade.^8^^,^ 16 ^,^ 19 Tais dados também foram observados na presente pesquisa. A indicação de parto cirúrgico convém às mulheres em risco obstétrico, entretanto, este vem sendo realizado com grande frequência entre gestantes de baixo risco, com maior renda e escolaridade, com sete ou mais consultas pré-natais e atendidas no serviço privado, expondo o recém-nato à iatrogenias decorrentes desse processo.^21^^-^ 23

Quanto às condições de nascimento dos menores de um ano de idade na presente pesquisa, a prematuridade ocorreu em ambos os extremos de idade materna e não apresentou associação estaticamente significativa. Ao contrário, alguns estudos apontaram que a adolescência tem 75% mais risco de ter parto prematuro que as mulheres adultas.^19^^,^ 24 Mas, outras investigações indicam que a maternidade tardia apresenta maior ocorrência de baixo peso ao nascer, parto pré-termo, asfixia neonatal, anomalias cromossômicas e macrossomias.^12^^,^ 13 Pesquisa realizada em Liverpool, com 9.506 registros de nascimentos, evidenciou maior prevalência de parto prematuro e baixo peso nos dois extremos de idade, mães adolescentes e com mais de 40 anos.^18^ O baixo peso ao nascer é um indicador isolado de morbimortalidade infantil^19^^,^ 22 ^,^ 25 e sua incidência quando comparada às mulheres adultas é mais que o dobro em adolescentes e duas vezes maior em mulheres com 35 anos ou mais.^8^

No presente estudo, poucas crianças receberam alta hospitalar após o nascimento e a maior proporção de mortes ocorreu nos primeiros 27 dias de vida. A mortalidade neonatal sugere causas oriundas especialmente da precária assistência ofertada às mães durante pré-natal e parto, bem como aos recém-nascidos em salas de parto e unidades neonatais. Nesses casos, há necessidade de estratégias no campo perinatal, com ações dirigidas ao desenvolvimento e fortalecimento dos serviços de saúde materno-infantil.^26^^,^ 27

Outra caracerística observada foi a predominância de afecções perinatais como causa do óbito, seguidas por anomalias congênitas e causas externas. Em pesquisa realizada no município de Cianorte, Paraná, entre 1999 a 2006, verificou-se que 80,7% das mortes infantis ocorreram no período neonatal, sendo 68,3% delas foram por causas perinatais.^12^ As altas proporções de óbito por afecções perinatais constituem sinal de alerta, pois podem indicar interrupção precoce da gravidez ou crescimento intra-uterino retardado, resultado de acompanhamento pré-natal inadequado.^21^^,^ 23 ^,^ 27 Quanto às anomalias congênitas, estudos apontam maior ocorrência entre mulheres com idade avançada,^7^^,^ 10 ^,^ 12 ^,^ 18 ^,^ 21 ^,^ 23 corroborando os achados deste estudo. Ao contrário, as causas externas foram mais evidentes nos filhos das adolescentes, fato preocupante por ser um evento totalmente prevenível e que pode estar associado ao despreparo da jovem mãe para cuidar do filho. 

A maioria dos óbitos infantis no Brasil pode ser considerada evitável, se garantido acesso correto e oportuno aos serviços de saúde. Por isso, investimentos em recursos humanos qualificados na assistência ao binômio mãe e filho em todas as instâncias assistenciais, assim como a presença estrutura física de atendimento hierarquizada e efetiva podem minimizar a ocorrências dessas mortes.^1^^,^ 21 ^,^ 23 ^-^ 25

Dessa forma, reafirma-se a necessidade de maiores investimentos na prevenção e promoção da saúde materna, principalmente nos extremos etários e, em especial, as adolescentes, buscando reduzir agravos gestacionais e mortes infantis evitáveis. Pesquisas longitudinais que trabalhem especificamente com extremos de idade materna devem ser estimuladas para melhor elucidação desses achados e contribuição com a literatura já existente.
